# Combination of the Manifold Dimensionality Reduction Methods with Least Squares Support vector machines for Classifying the Species of Sorghum Seeds

**DOI:** 10.1038/srep19917

**Published:** 2016-01-28

**Authors:** Y. M. Chen, P. Lin, J. Q. He, Y. He, X.L. Li

**Affiliations:** 1 College of Electrical Engineering, Yancheng Institute of Technology, No.1 Middle Road Hope Avenue, Yancheng, Jiangsu Province 224051, P.R. China; 2 College of Biosystems Engineering and Food Science, Zhejiang University, 866 Yuhangtang Road, Hangzhou 310058, China

## Abstract

This study was carried out for rapid and noninvasive determination of the class of sorghum species by using the manifold dimensionality reduction (MDR) method and the nonlinear regression method of least squares support vector machines (LS-SVM) combing with the mid-infrared spectroscopy (MIRS) techniques. The methods of Durbin and Run test of augmented partial residual plot (APaRP) were performed to diagnose the nonlinearity of the raw spectral data. The nonlinear MDR methods of isometric feature mapping (ISOMAP), local linear embedding, laplacian eigenmaps and local tangent space alignment, as well as the linear MDR methods of principle component analysis and metric multidimensional scaling were employed to extract the feature variables. The extracted characteristic variables were utilized as the input of LS-SVM and established the relationship between the spectra and the target attributes. The mean average precision (MAP) scores and prediction accuracy were respectively used to evaluate the performance of models. The prediction results showed that the ISOMAP-LS-SVM model obtained the best classification performance, where the MAP scores and prediction accuracy were 0.947 and 92.86%, respectively. It can be concluded that the ISOMAP-LS-SVM model combined with the MIRS technique has the potential of classifying the species of sorghum in a reasonable accuracy.

Sorghum is a diverse genus consisting of both cultivated and wild species. Most of them have considerable genetic and morphological diversity^1^. Sorghum halepense (Johnsongrass) is an extremely invasive noxious weed which is considered to be one of the ten worst weeds in the world^2^. Silk sorghum resembles Johnsongrass and is also treated as a dangerous weed because of its aggressive competition with crop plants for soil nutrients, water, space, and frequent toxicity to grazing stock. In contrast to these weeds in sorghum, the species of S.sudanense and S.propinguum are the cultivated pasture plants.

The species of S.halepense, Silk sorghum, S.sudanense and S.propinguurn are belonging to Eu-sorghum section. S.halepense and Silk sorghum are considered as the dangerous weeds which have been forbidened in China; meanwhile, Silk sorghum is also forbidened because one of its parent is S.halepense. The similarities in morphology and cytology make it difficult to classify the dangerous species, therefore the classification has to be done correctly and quickly^3^,^4^. Morphological characters have traditionally been used to distinguish them. Molecular genetics, particularly in the area of gene sequencing, have provided an additional source of data for the systematic studies of genetic relatedness^5^. These methods are labor-intensive, environment-contaminated and destructive, and not available for real-time measurement and control.

The method of combination of the infrared spectroscopy technique and chemometrics is considered as a promising detecting technique, which can be used to rapidly and nondestructively classify the substances. Neirivaldo *et al.* presented the infrared spectroscopy technique as a rapid and nondestructive methodology to determine the origin of gasoline^6^. Guo *et al.* discriminated the maize seed varieties based on the near infrared spectroscopy^7^. Geng *et al.* monitored the viability of soybean seed by employing fourier transform near-infrared (FT-NIR) spectroscopy^8^. Liu *et al.* geographically classified the Spanish and Australian Tempranillo red wines by the visible and near-infrared spectroscopy^9^. Chen *et al.* classified the vinegar quality according to the total acid content through the near-infrared spectroscopy techniques and the nonlinear regression methods and obtained a satisfying result^10^.

The manifold learning algorithms have been used for the dimensionality reduction (DR) of the high-dimensional spectral data, and provide the important means for the feature extraction^11^,^12^. The manifold learning algorithms can be divided into two categories: one is the linear methods such as PCA, LDA and LPP, and the other is the nonlinear methods such as isometric mapping (ISOMAP), local linear embedding (LLE), laplacian eigenmaps (LE) and local tangent space alignment (LTSA). The traditional linear manifold DR methods assume that the structures of dataset are essentially linearly correlated. These methods can effectively learn a linear structure when the variables are totally linearly correlated. But when the structure of data is highly nonlinear, the traditional method has difficulty expressing the real structure of the data set. In this paper, both of the linear and nonlinear manifold DR methods were conducted to approximate the high dimensional spectral data and their performances were compared.

The purpose of this study was to assess the genetic relationships among S.halepense, Silk sorghum, S.sudanense and S.propinguum on the basis of middle infrared spectroscopy (MIRS) technique and chemometrics, and provide the evidence for the classification among the four species. The paper was organized as following: Firstly, the materials and equipment for acquiring spectra were introduced. Secondly, the chemometrics methods for preprocessing and modeling were presented. Thirdly, an illustrative example of detecting the nonlinearity in the sorghum spectra was provided. Fourthly, the linear and nonlinear manifold DR methods which were used to extract the feature variables were discussed. The prediction performance using the extracted feature variables and LS-SVM regression method were compared. Finally, the conclusions were drawn.

## Materials and Methods

### Materials

Four species of sorghum used for the experiment were collected from the regions of China, America and Argentina respectively. Their primary information were listed in Table 1.

In this study, the Fourier Transform middle Infrared Spectrometer (Japan) of JASCO Model FT/IR-4000 was used to capture the reflectance spetrum of the samples. The range of spectra was from 7000 to 350 cm^−1^ and the resolution was set as 4 cm^−1^. The samples were measured in a quartz cuvette that was a standard accessory of this spectrophotometer. The cuvette was washed with distilled water when each sample was finished. Each observation was scanned 30 times, and the 30 spectral data were taken average as the reflectance to represent each sample. The software of Spectra Manager CFR was used for spectral data acquisition and analysis. The temperature was kept at about 25 °C during the whole experiment.

## Chemometrics Methods

In this section, two quantitative statistical methods including the Durbin–Watson test and the Runs test and the augmented partial residual plot (APaRP) were used to diagnose the nonlinearity of high-dimensional spectral data. Subsequently, four kinds of nonlinear DR methods of ISOMAP, LLE, LE and LTSA were presented. Finally, the nonlinear regression method of LS-SVM for modelling and forecasting was introduced.

### Durbin–Watson and Runs test

The Durbin–Watson test examines the null hypothesis that there is no correlation between the successive residuals and the alternative hypothesis that the correlation exists. To estimate the null hypothesis, statistic 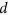 is computed as following:


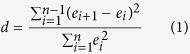


Two critical parameters of 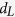 and 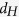 are used to analyze the correlation of the residuals 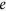. When 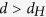 the hypothesis can be accepted, which indicate there are uncorrelation in residuals and nonlinearity in model; When 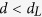 the null hypothesis is rejected and there are correlation in residuals and linearity in model. In other cases, the test is inconclusive.

The Runs test uses a test statistic (*z*) which is approximately normally distributed when the null hypothesis is true. The desired test statistic is the difference between the number of runs (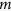) and its mean (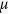) and divided by its standard deviation (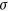) as following:


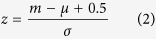


Where 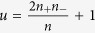, 
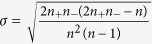
 and 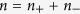. The 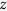-value tables show the probability to reach 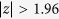 is 0.05. When the test value is larger than that value, the model is considered as the nonlinearity^13^,^14^.

### Augmented partial residual plot

The augmented partial residual plot can be used to diagnose the nonlinearity of the system^15^. APaRP correlates the first 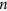 principle components (PCs) of the predictor 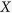 and the square of the first PC with the response 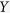:


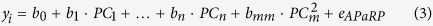


Where 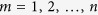 and 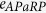 is the fitting residual. The diagnostic figure is obtained by plotting the sum 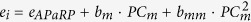 against the 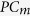.

### Isometric feature Mapping

Isometric feature mapping (ISOMAP) represents a generalization of metric multidimensional scaling (MDS) to nonlinear manifolds^16^. Unlike classical MDS which attempts to preserve the Euclidean distance between the data points, ISOMAP finds an embedding in which the geodesic distance between two points in the input space is as close as possible to the Euclidean distance between their projections in the target space^17^.

Let 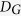 denotes the matrix of the geodesic distances between the points in the neighborhood graph. The embedding into the d-dimensional space is computed by minimizing the function:


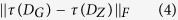


where 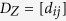 is the matrix of pairwise Euclidean distances 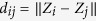 of the data projections in 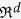, the 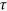 operator converts the distances to the inner products and 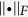 denotes the Frobenius norm of a matrix. The global minimum of Eq. (4) is achieved by computing the 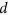 eigenvectors associated to the 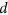 largest eigenvalues of the geodesic distances matrix 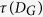.

### Locally linear embedding

Locally linear embedding (LLE) attempts to recover the global structure of nonlinear manifolds from locally linear fits, so as to preserve the local geometry of the input data in the low-dimensional space^18^,^19^. Once the neighborhood graph is constructed based on the Euclidean distance, LLE represents each point 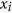 as a linear combination of its neighbors:


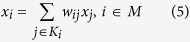


Where 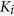 is the set of indices of the 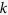 nearest neighbors of 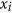, and the generic weight 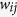 highlights the role of neighbor 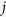 in the reconstruction of point 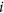. The weight coefficients for all data points can be computed by minimizing the function:


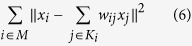


Which is subjected to the constraints 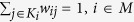, thus the weights are invariant to translations, rotations and rescales of individual points and their neighbors.

### Laplacian eigenmaps

Laplacian eigenmaps (LE) resorts to the notion of the Laplacian of the neighborhood graph in order to compute low-dimensional projections^20^. In LE the edge of the neighborhood graph which connecting point 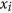 to one of its nearest neighbors 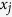 is weighted according to two alternative criterions: the weight 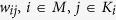 and the projection 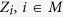. 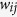 is computed by the heat kernel method, using the Gaussian kernel function:


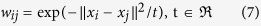


which assigns an increasing weight as the points 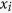 and 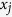 tend to be closer. The simple-minded approach sets instead 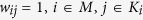. In both cases, 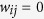 for 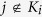.

The projections 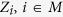 of the data points in the reduced space are then computed by minimizing the function:


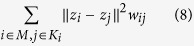


Which entails a heavy penalty for neighboring points mapped far apart.

By introducing the Laplacian matrix 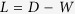 of the neighborhood graph, where 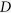 is a diagonal matrix with entries 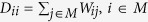, the minimization of Eq. (8) reduces to the following optimization problem:


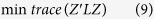


The solution of Eq. (9) is obtained in closed form by computing the 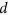 eigenvectors corresponding to the 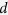 smallest nonzero eigenvalues of the generalized eigenvalue problem 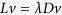, and by setting the projections 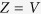.

### Local Tangent Space Alignment

The Local Tangent Space Alignment algorithm (LTSA)^21^ is a nonlinear dimensionality reduction method that aims to find a global coordinate system within a low dimensional space that best characterises the high dimensional data set. LTSA finds the 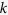 nearest neighbors of each data point 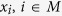, and builds the centered matrix of neighbors 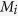 which includes also 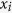. Thereafter, it approximates the 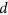-dimensional tangent space 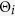 of each neighborhood by computing the first 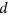 right singular vectors of 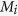 corresponding to its 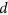 largest singular values^22^. The performance of LTSA highly depended on the quality of the local tangent spaces approximation, which means that if the data points were not exactly located on a two-dimensional surface, this approximation would be very poor. Thereby, before implementing the LSTA method to extract the intrinsic spectral variables, the original spectral data set was preprocessed in order to reduce noise for further constructing the approximate smoothing manifold surface^12^. Local tangent space alignment has been successfully applied to microarray data analysis^23^ and face recognition^24^.

### Least squares support vector machine

LS-SVM establishes the regression model by a nonlinear mapping function 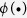, the input variables are mapped into high dimensional feature space^25^,^26^. Then convert the optimization problem into a constrained condition. Using Lagrange multipliers to solve optimization problems and solve partial differential of variables. According to Mercer condition, existing mapping function 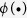 and the kernel function 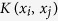:


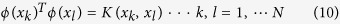


The popular kernel functions include linear kernel function, polynomial kernel function, radial basis function (RBF) kernel function^27^ and multilayer perceptron kernel function. In this paper the RBF kernel function is used to obtain the function of LS-SVM estimation:


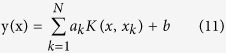


## Results and Discussion

In this section, the nonlinearity of sorghum MIRS was investigated. Six illustrative examples of different DR methods were shown. The LS-SVM regression methods for modelling and forecasting were discussed. There were 180 sorghum observations used for the analysis. The samples were randomly divided into two sets including the training and the test set. The training set consisted of 140 samples, where each class consisted of 35 samples. The rest of 40 samples were used for the validation.

### Reflectance spectral investigation

**Figure 1:**
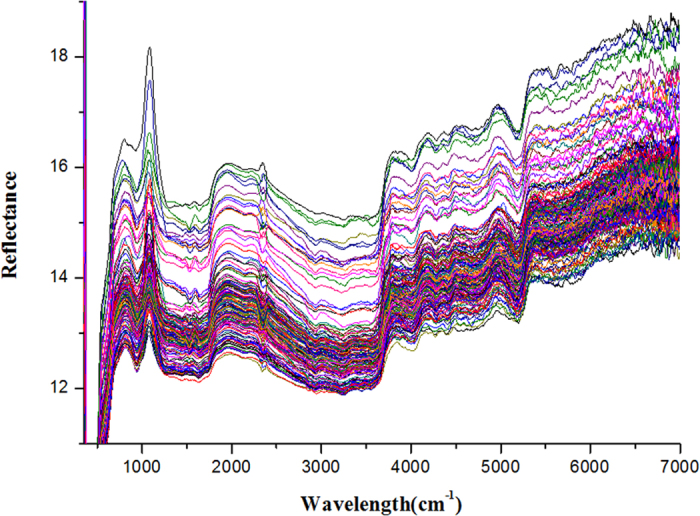
Average reflectance spectra of 180 sorghum samples between the wavelengths of 7000-350 cm^−1^.

The spectral reflectance curves of the different sorghum seeds were plotted in Fig. 1. As shown in Fig. 1 the differences between the spectra of four kinds of sorghum seeds were extremely small throughout the whole measurement area. The trend of different spectral curves was similar which indicated that the test instance belonged to the similar species. It is hard for us to directly discriminate the species from the spectral curves. There were consistent baseline shifts and bias in the spectra due to the effect of light scattering or the length of light path etc. The peaks and valleys on the spectral curves are related to some special elements in sorghum seeds. The reflectance intensity of characteristic peaks is different because of the diverse content of components in each sample. These characteristic information will be used for the subsequent discrimination target.

### Pretreatments

Spectral preprocess is a preliminary step in the chemometric analysis, which is usually performed prior to the model calibration. The goal of preprocess is to reduce the effects of length variation of light path, light scattering and to enlarge the hidden information in the original spectral data^28^. In this work, the pretreatment technique of direct orthogonal signal correction (DOSC) was used to deal with the raw sorghum spectral data. Before performing the DOSC algorithm, two crucial parameters including the number of DOSC components and the tolerance factor should be determined. The number of the DOSC components should not be set too large, since that the large number of components would likely lead to overfitting phenomenon^29^. The optimal number of DOSC components was determined in terms of the number of largest magnitude eigenvectors of inner product space in the orthogonal subspace, which corresponded to the largest eigenvalues^30^. The tolerance was used to determine the number of singular values by calculating the generalized Moore–Penrose pseudoinverse of spectra. In this study, the DOSC components and the tolerance were set to be 6 and 0.001, respectively. So far, there has no systematic methodology for estimating the optimal parameters of DOSC components and tolerance, so the optimal values were determined after several values were tried.

### Detecting nonlinearity

**Figure 2:**
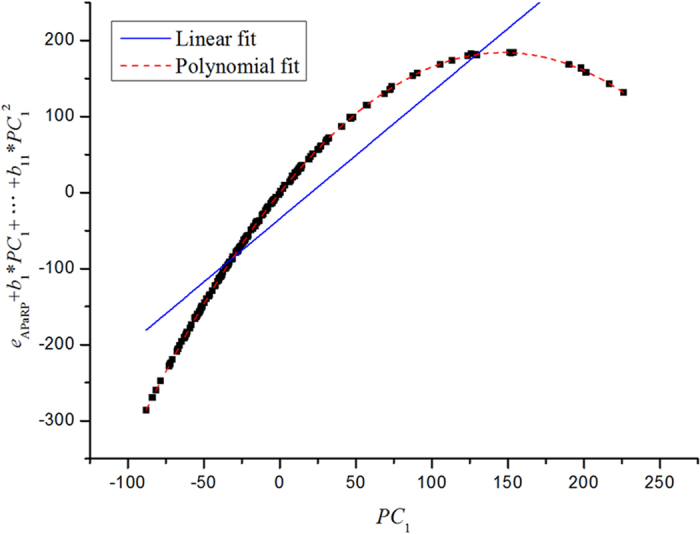
Detecting nonlinearity in the sorghum dataset using Augmented partial residual plot (APaRP) for 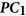, where the first eleven PCs are included in the model.

Two quantitative statistical methods of the Durbin–Watson test and the Run test were performed to check the nonlinearity of model. The Durbin–Watson test value 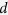 was 2.376 which was greater than 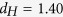, and the Runs test value of 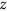 was 3.296 which was larger than 1.96. These test results illustrated that there were significant nonlinearity in the sorghum data set. Furthermore, the APaRP method was used to determine whether there was nonlinearity in the sorghum MIRS data. The results of polynomial fitting shown in Fig. 2 illustrated that there were significant nonlinearities in the sorghum data set.

### Feature variable extraction

In this section, six different manifold DR methods of ISOMAP, LLE, LE, LTSA, PCA and MDS were used to extract the feature spectra from the full spectral data; then, the performance of them were compared.

The intrinsic dimensionality 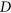 of the manifold and the most promising parameters of each method were determined according to the accuracy evaluation. The estimate of 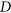 was still an open question, for which no dominant techniques currently exist. In this work, the parameters of each method were optimized by maximizing the prediction accuracy of LS-SVM regression process of the training set within a preliminary 5-fold cross-validation run. Specifically, the number of nearest neighbors 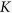 used for building the neighborhood graph was varied in the interval^2^,^15^ with an incremental step size of 1. For weighting the edges of the neighborhood graph in LE, both of the simple-minded and the heat kernel approaches were tested with 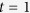. Finally, the heat kernel rule provided the best results and was used for the further estimations.

**Figure 3:**
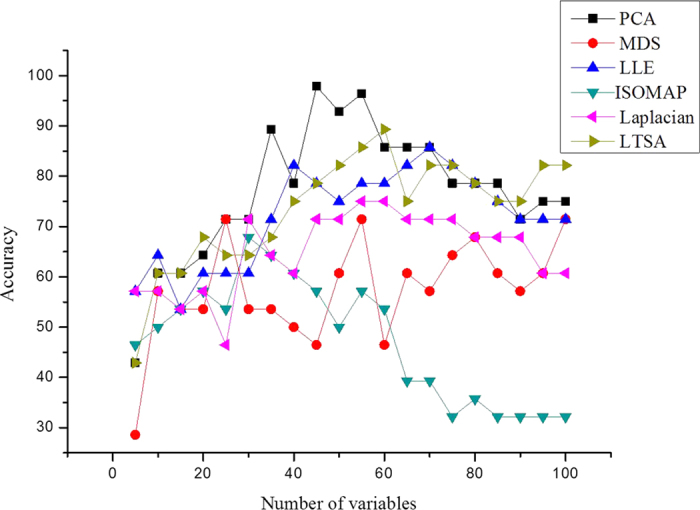
Prediction accuracy of training set with different numbers of variables.

Figure 3 shows the prediction accuracy of using the different values of optimal parameter. The highest prediction accuracy of training set using LE-LS-SVM method was 75% when 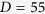 and 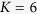. The LLE method attained the highest prediction accuracy of 85.71% when 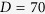 and 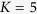. The highest prediction accuracy of training set by the ISOMAP based method reached 97.84% with 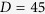 and 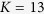. The LTSA method attained the highest prediction accuracy of 89.26% when 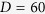 and 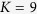. For the linear manifold DR methods, the PCA obtained the highest prediction accuracy of 67.85% with 30 eigenvalues while the MDS method reached the highest prediction accuracy of 71.43% with 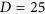.

The prediction effect of ISOMAP algorithm turned out to be the best, followed by the algorithms of LTSA LLE, LE, PCA and MDS. It could be concluded that the nonlinear methods of DR obtained the superior prediction performance (see table 2).

The parameters of 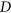 and 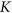 used for predicting test set were determined by the highest accuracy of training set. The specific values of 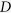 and 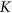 were showed in Table 2. It could be seen that the three different kinds of nonlinear manifold DR methods obtained the highest prediction accuracy with more than 45 feature variables, while the number of feature variables of two linear manifold method is less than 30. In this study, linear manifold methods extract less characteristic variables than nonlinear methods. This might because that the nonlinear DR methods extract the principal components via mapping all the variables into the high dimensional feature space, in which the characteristic variables could be fully used. However, some nonlinear characteristic variables cannot be taken into account in the original linear spaces by using the linear based methods. Therefore, the number of feature variables extracted by the linear based algorithms is less than the nonlinear ones.

### Comparing prediction results of LS-SVM models with different manifold learning methods

In this section, the nonlinear model of LS-SVM was used to distinguish the sorghum samples and the performance of handling nonlinearity was discussed. The LS-SVM algorithm could be used to transform the data from the original space into the high-dimensional feature space via kernel function. The commonly used kernel functions included linear kernel, polynomial kernel and Radial basis function (RBF) kernel. The RBF kernel generally showed the optimal performance in handling the nonlinear relationship between the spectra and target attributes and could be used to reduce the complexity of computation during the training procedure^31^. Thus, RBF kernel was selected as the kernel function for the LS-SVM model in this paper. Two important parameters of gamma (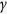) and square of sigma (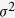) need to be optimized in the RBF kernel function. A two-step grid search technique using geometric steps with leave-one-out cross validation was used to obtain these two optimal parameters ranging from 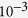 and 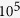
32,33. The first step of grid search was a crude search with a large step size and the second search was a specified search with a small step size. After the determination of these two important parameters, the performance of LS-SVM model was evaluated by forty validation samples with parameters of 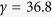 and 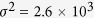.

**Figure 4:**
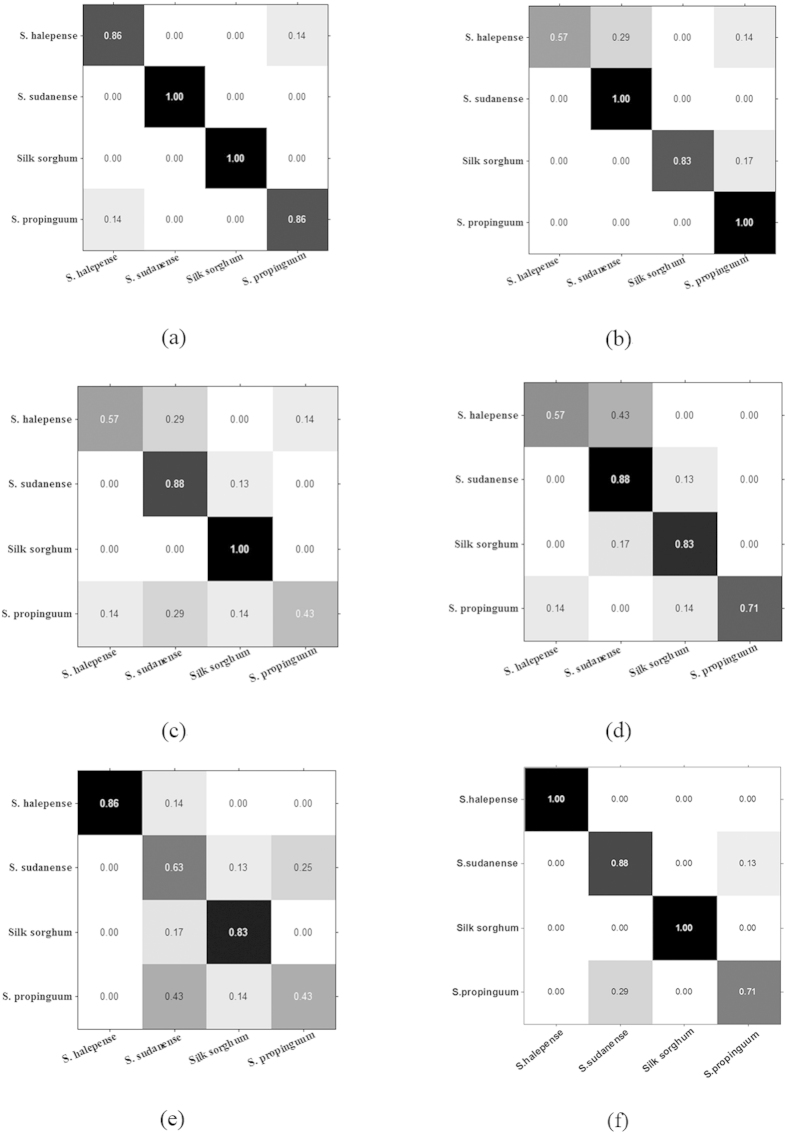
Confusion matrices of prediction results of LS-SVM by different manifold dimensionality reduction methods of (a) ISOMAP, (b) LLE, (c) LE, (d) MDS, (e) PCA and (f) LTSA.

The precision-recall (PR) curves, mean average precision (MAP) scores, prediction accuracy and confusion matrices (CM) were respectively used to grade the prediction performance of models. These parameters and charts were widely used to measure the performance of learning algorithm. For the CM, each column represented the instances in a predicted class, while each row represented the instances in an actual class. As shown in Fig. 4(a) the ISOMAP-based system can make the distinction between S.sudanense and Silk sorghum with other type pretty well, and all the unknown samples were correctly predicted. For S.halepense and S.propinguum, only 14% of the unknown samples were mis-classified. Figure 4(b) illustrates the CM of LS-SVM model by LLE method, it was shown that all the S.sudanense and S.propinguum samples were correctly distinguished with others, which illustrated a perfect prediction effect. For Silk sorghum, 17% unknown samples were misjudged as S.propinguum, and for S.halepense the system had trouble distinguishing between the seeds of S.halepense and S.sudanense. Figure 4(c) shows the CM of LE method, the result illustrated that the system could make the perfect distinction between the Silk sorghum and other types, moreover, the distinction between the S.sudanense and other types also good enough. But the system cannot well distinguish S.halepense and S.propinguum with S.sudanense. Figure 4(d) shows the CM of MDS method, it illustrated that the system can distinguish S.sudanense and Silk sorghum from others, except for the species of S.halepense. About half of the S.halepense samples were misjudged as S.sudanense. For S.propinguum, 14% of the unknown samples were mis-distinguished as S.halepense and Silk sorghum respectively. Figure 4(e) shows that PCA could be used to distinguish S.halepense and Silk sorghum from others, but has trouble distinguishing the seeds of S.sudanense and S.propinguum with others. Figure 4(f) shows that LTSA could accurately distinguish S.halepense and Silk sorghum with others, but have some difficulties in discriminating S.sudanense with S.propinguum.

**Figure 5:**
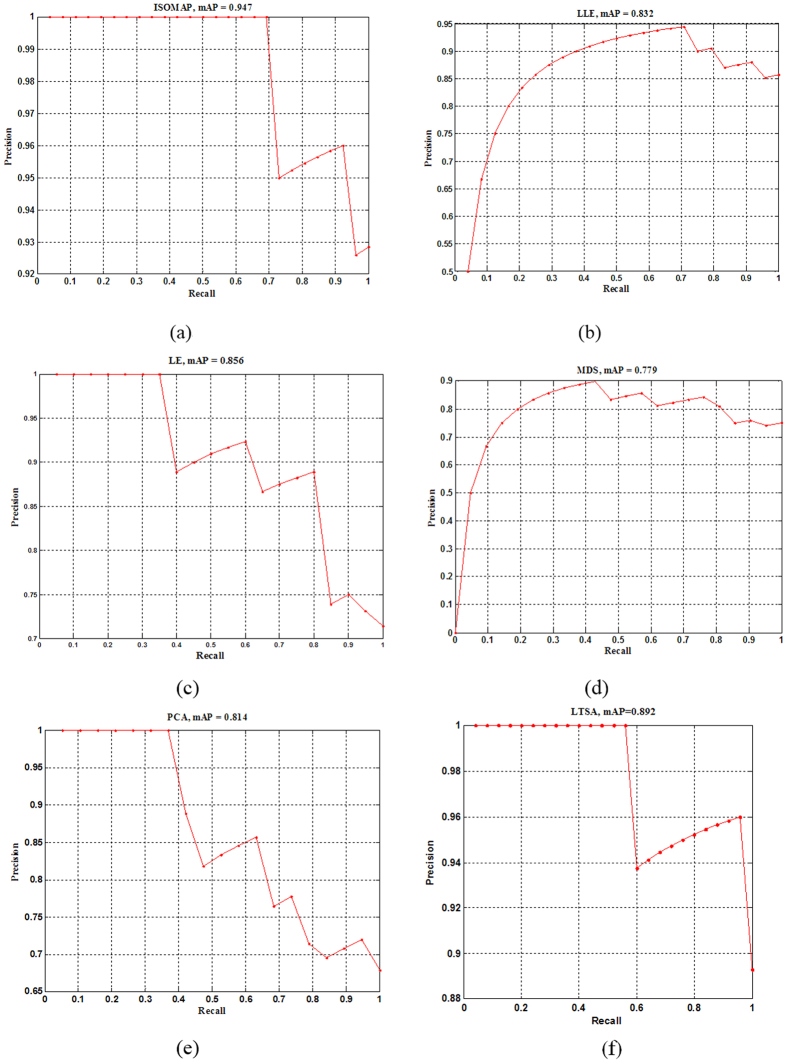
Precision-recall (PR) curves of prediction results of LS-SVM by different manifold dimensionality reduction methods of (a) ISOMAP, (b) LLE, (c) LE, (d) MDS, (e) PCA and (f) LTSA.

The MAP score was also used to measure the overall prediction precision of model. Generally, a good model should gain a high MAP score and a large area surrounded by the PR curve. The PR curves of different LS-SVM models using different manifold methods were shown in Fig. 5. The algorithm of ISOMAP got the highest MAP score of 0.947, and followed by LTSA of 0.892, LE of 0.856, LLE of 0.832, PCA of 0.814 and MDS of 0.779, respectively.

The accuracy of prediction was shown in Table 2. The prediction accuracy of three nonlinear manifold methods were all above 75%. Moreover, the ISOMAP method reached the highest value of 92.86% which demonstrated a good performance. For the two linear manifold methods, the prediction accuracy of PCA method became the lowest of 67.86% while the value for the MDS method was 71.43%. Both of them were below 75%. The results indicated that the nonlinear MDR method combined with LS-SVM regression method demonstrated better performance than the linear manifold DR methods. The reason might be that the input variables of the data sets had the strong nonlinear structure. The linear-based methods failed to model the nonlinear correlation structure properly.

## Conclusions

In this paper the manifold dimensionality reduction methods and nonlinear regression model of LS-SVM combined with MIRS techniques were presented for detecting the species of sorghum seeds. The nonlinearity of the raw spectral data was detected by the method of Durbin test, run test and APaRP. The nonlinear MDR methods of ISOMAP, LLE, LE and LTSA and linear manifold DR methods of PCA and MDS were utilized to extract characteristic variables. The extracted feature variables were used as the input of LS-SVM regression model for prediction of the class of unknown sorghum samples. The performance of prediction models were evaluated by CM, PR curves, MAP scores and prediction accuracy respectively. The ISOMAP-LS-SVM model obtained the highest MAP scores and prediction accuracy of 0.947 and 92.86% respectively. The operation of spectroscopy-based measurement is much simpler and more convenient than the traditional physical and chemical methods. Thereby, the investigation provided a superior alternative method to rapidly and accurately classify the species of sorghum seeds.

## Additional Information

**How to cite this article**: Chen, Y. M. *et al.* Combination of the Manifold Dimensionality Reduction Methods with Least Squares Support Vector Machines for Classifying the Species of Sorghum Seeds. *Sci. Rep.* **6**, 19917; doi: 10.1038/srep19917 (2016).

## Figures and Tables

**Table 1:** The primary information of four kinds of sorghum species.

Taxa	Belongings	Locality
S.halepense	Dangerous weed	America
Silk sorghum	Dangerous weed	Argentina
S.sudanense	Pasture plant	America
S.propinguum	Pasture plant	China

**Table 2:** Prediction parameters and accuracies estimated by using different manifold dimensionality reduction methods.

Manifold dimension reduction method	Dimension	K nearest neighbor	Accuracy of prediction model (%)	
Nonlinear	LLE	70	5	85.71
	ISOMAP	45	13	92.86
	LTSA	60	9	89.26
	LE	55	6	75.00
Linear	PCA	30	—	67.86
	MDS	25	—	71.43
